# Supplementary material for: Mouse models of Loa loa
Source: Nat Commun. 2019 Mar 29;10:1429. doi: 10.1038/s41467-019-09442-0 (PMC6441053; doi:10.1038/s41467-019-09442-0)
Supplement: Supplementary file 1 — Supplementary Information [file 41467_2019_9442_MOESM1_ESM.docx]

Mouse models of *Loa loa*

Pionnier et al.

*Supplementary Methods*

Recovered female and male worms’ lengths were measured using an Olympus BX60 microscope.

Female *L. loa* worms were processed for embryograms. Briefly, each female was minced into 1mm pieces using a scalpel and transferred to a sterile mortar containing 1mL of phosphate buffered saline (PBS). After complete homogenization, 15µL of the homogenate was loaded on a Neubauer counting chamber and the embryonic stages (morulae, coiled microfilariae, stretched microfilariae and oocysts) were counted.

Female worms were also considered for immunohistochemistry analyses to confirm observations seen on whole mounts. In short female worms were fixed in hot 70% ethanol, embedded in paraffin and then cut in 4µm sections which were stained with hematoxylin-eosin. Samples were analysed on an Olympus BX60 microscope.

*In vitro* drug screening assays were performed on *Brugia malayi* and *Loa loa* mf. *B. malayi* mf were obtained and purified from peritoneal washings of experimentally infected *Meriones unguiculatus* gerbils as previously described^1^. Briefly, drugs (Sigma) were prepared in 1% DMSO and were tested for 5 days on approximately 20-30 mf per well on 96-well plates (3 wells per drug concentration). Parasites survival was calculated every day by assessing mf motility under the microscope. Plates were incubated at 37°C 5% CO_2_ for the whole duration of the assay. An MTT test (Sigma) was also performed as previously described^2^ after 5 days in vitro incubation with the drugs on 5000 mf per well and absorbance was read 590nm.

*Supplementary references*

1. Halliday, A. *et al.* A murine macrofilaricide pre-clinical screening model for onchocerciasis and lymphatic filariasis. *Parasit Vectors* 7, 472 (2014).
2. Sjoberg, H. *et al.* Short-course, oral flubendazole does not mediate significant efficacy against Onchocerca adult male worms or Brugia microfilariae in murine infection models. *PLoS Negl Trop Dis* 13(1): e0006356 (2019).

*Supplementary Figures*

**A**

**B**

**♂**

♀

♀


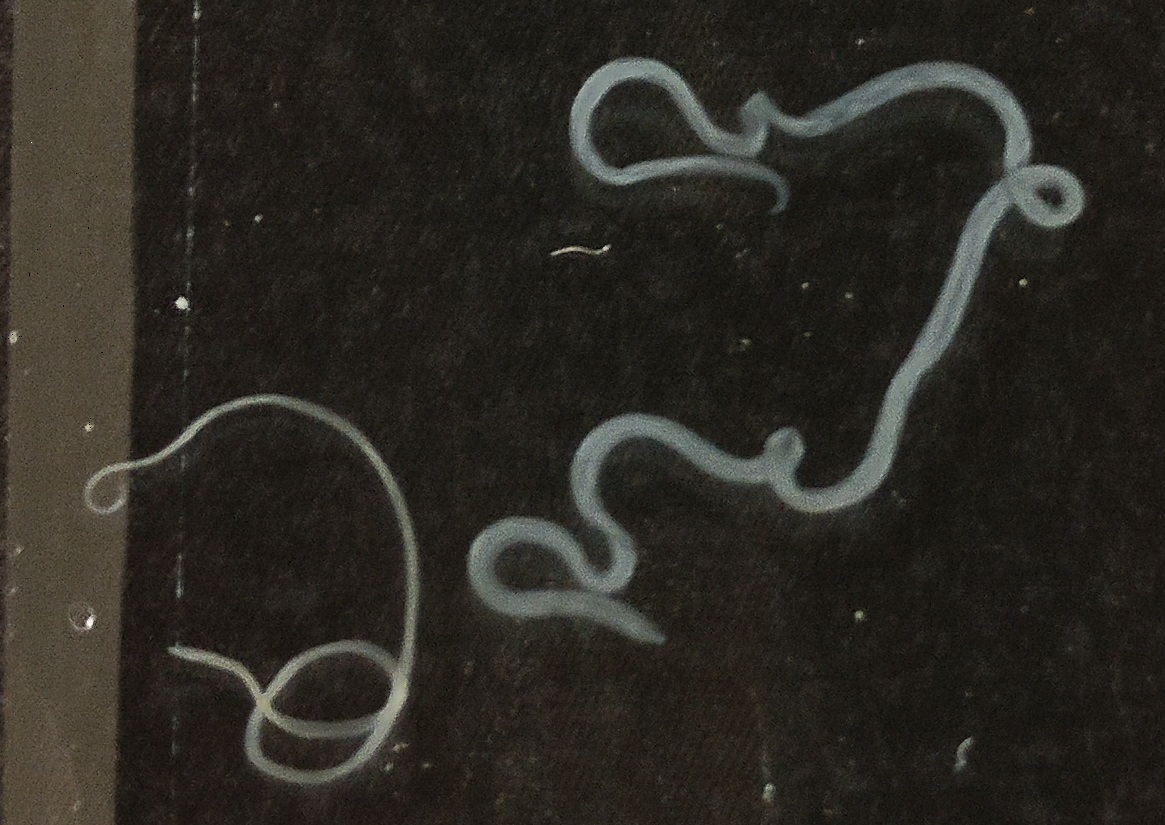


**♂**

**♀♀**

**C**

Supplementary Figure 1: Recovered *L. loa* adult worms from mice 5 months post-infection were viable and fully mature. (A) representative picture of *Loa loa* female and male worms recovered from an infected BALB/c Rag2^-/-^γc^-/-^ mouse, 5 months post-infection. Scale bar = 0.5cm. (B) female and male worm length measured on a representative subsample at readout 5 months post-infection, n=12, mean +/- SEM, T test ***: P<0.001 (C) comparative cardiopulmonary microfilaremia production 5 months post-infection in selected lymphopenic mouse strains, n=5-8, mean +/- SEM.


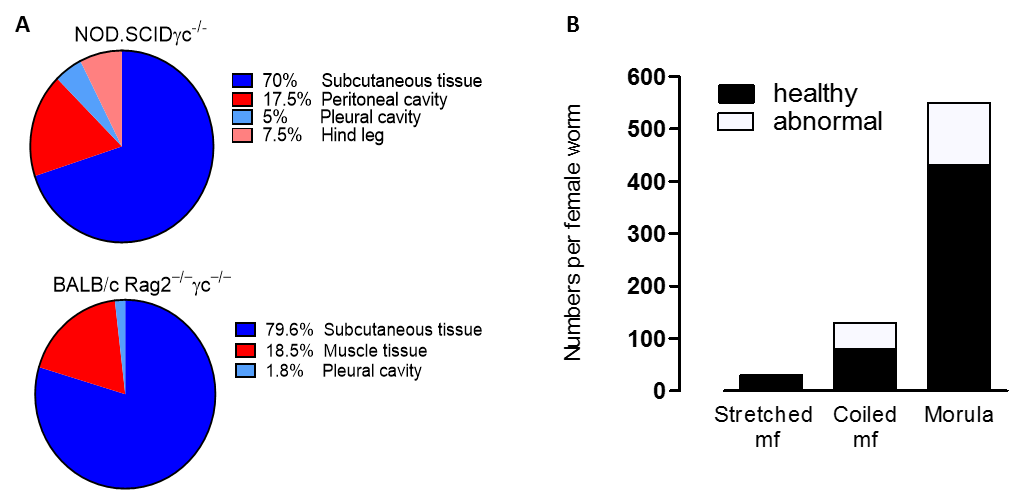


Supplementary Figure 2: Recovered *L. loa* adult worms 1 month post-implantation were still viable and fully mature. (A) tissue distributions of adult *L. loa* in NOD.SCIDγc^-/-^ or BALB/c RAG2^-/-^γc^-/-^ mice 1-month post-infection, n=5. (B) embryogram outcome from *L. loa* females recovered from RAG2^-/-^ mice implanted with *L. loa* adults and culled 1 month post-implant. Uterine content is expressed as the total number per female on average of stretched mf, coiled mf and morula embryonics stages depending on their healthy status, n=10.


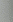

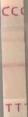

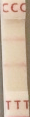


C

T


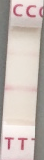

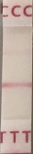

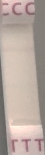

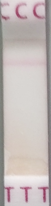


1

2

3

4

5

6

Supplementary Figure 3: Murine *L. loa* status of infection can be detected through FTS. Representative FTS outcomes depending on tested samples; test being positive if test band (T) appears concomitantly with the control (C) band. 1: BALB/c RAG2^-/-^ mouse serum post adult implant, 2: BALB/c Rag2^-/-^γc^-/-^ mouse serum 5 months post- L3 infection, 3: BALB/c Rag2^-/-^γc^-/-^ mouse serum 3 months post- L3 infection, 4: *L. loa* female *in vitro* culture supernatant, 5: serum from *L. loa* mf infused SCID mouse, 6: serum from naïve BALB/c mouse.

Supplementary Figure 4: Microfilaraemias are not further increased in infused splenectomised SCID mice compared with SCID controls. comparative cardiac (A) and peripheral (B) microfilaraemias between splenectomised and non-splenectomised CB.17 SCID mice. Peripheral microfilaraemias were measured at 2, 4, 6 and 8 dpi whilst cardiac microfilaraemias were determined at 8dpi readout. n=8-11, nd = not different, independent T-tests of log-transformed data at indicated timepoints.

Supplementary Figure 5: *In vitro* drug screening assays against blood borne microfilariae do not reflect *in vivo* drug sensitivities to macrocyclic lactones *Brugia malayi* (Bm) or *Loa loa* (Ll) mf survival on 5-days *in vitro* screening using ivermectin (A, B) and moxidectin (C, D) drugs at a range of 1-1000x C_maxs_ (40ng/mL). Photometric absorbance after MTT assays on *Brugia malayi* (E) and *Loa loa* (F) mf after 5 days *in vitro* culture with either ivermectin (IVM), moxidectin (MOX) or DMSO control. One-way ANOVA with Bonferroni post-hoc test, *: P<0.05, ***: P<0.001, ****: P<0.0001.

*
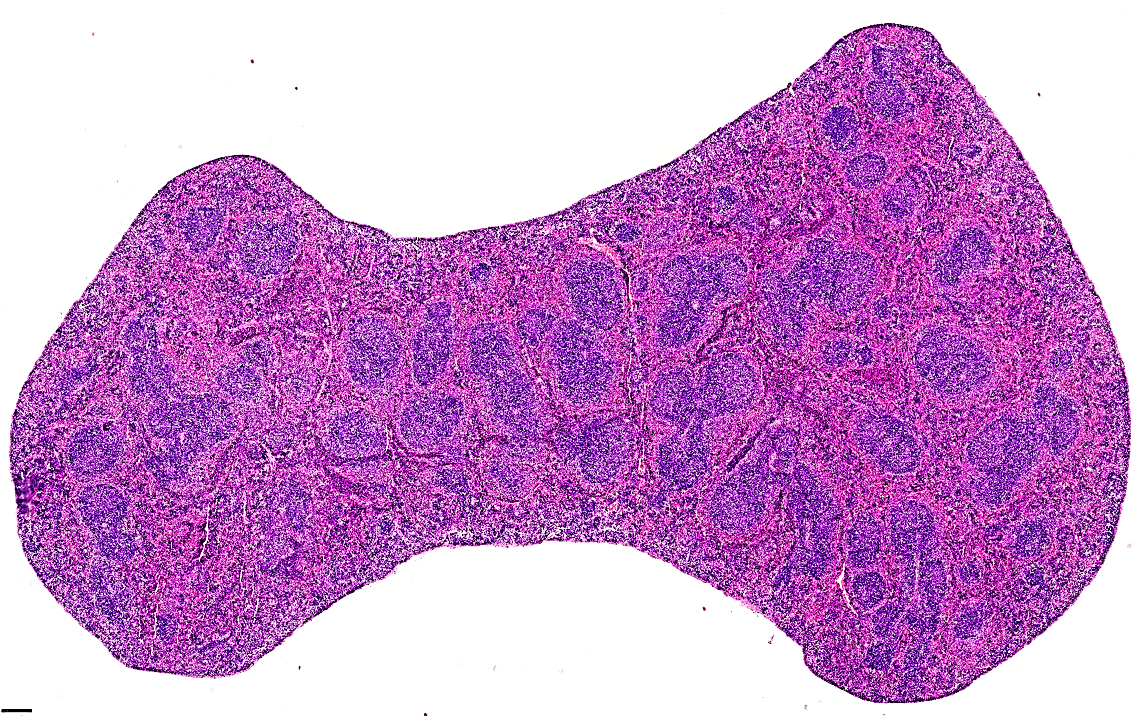
*A

*
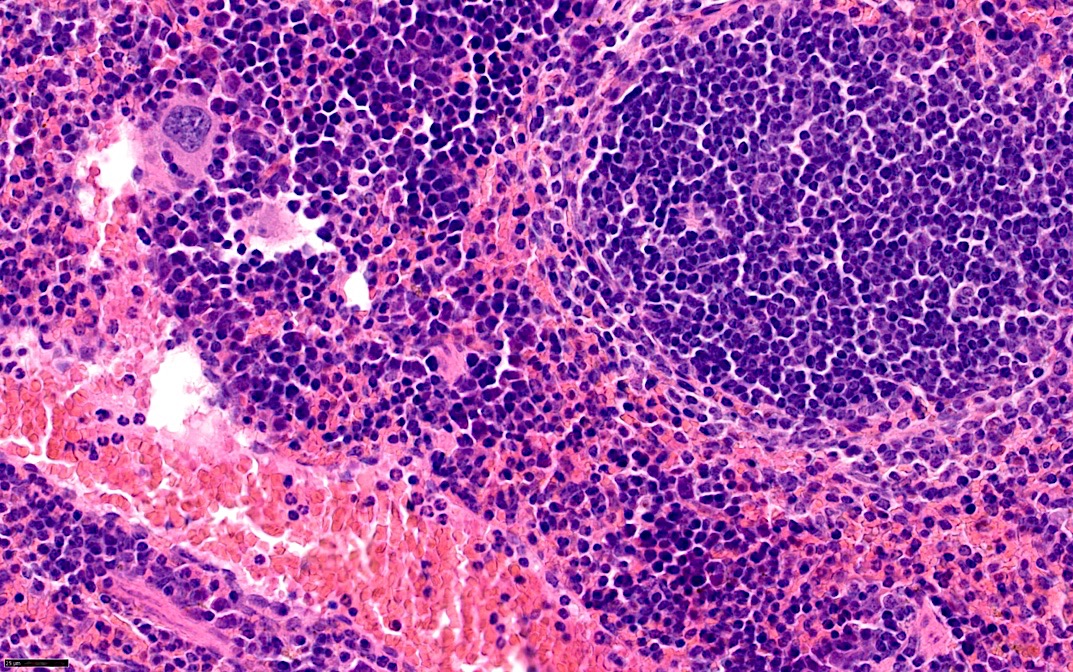
*B

**WP**

**V**

**MZ**

**WP**

**V**

**RP**

Supplementary Figure 6: Ivermectin treatment induces splenic eosinophilia in *L. loa* microfilaraemic mice. (A) representative hematoxylin eosin histological section of a spleen derived from a BALB/c mouse 7 days post-IVM 5mg/kg treatment following immune-priming with s.c. inoculation of 1x10^4^ heat-killed *L. loa* mf and infusion with 40,000 purified *L. loa* mf. Scale 250µm. (B) Zoomed section illustrating the marginal zone (MZ), the red pulp (RP), the white pulp (WP) and vasculature (V) An eosinophilic infiltrate (white arrows) is evident within vasculature, proximal to vessel walls and can also be detected in the red pulp (white arrows), scale 25µm.


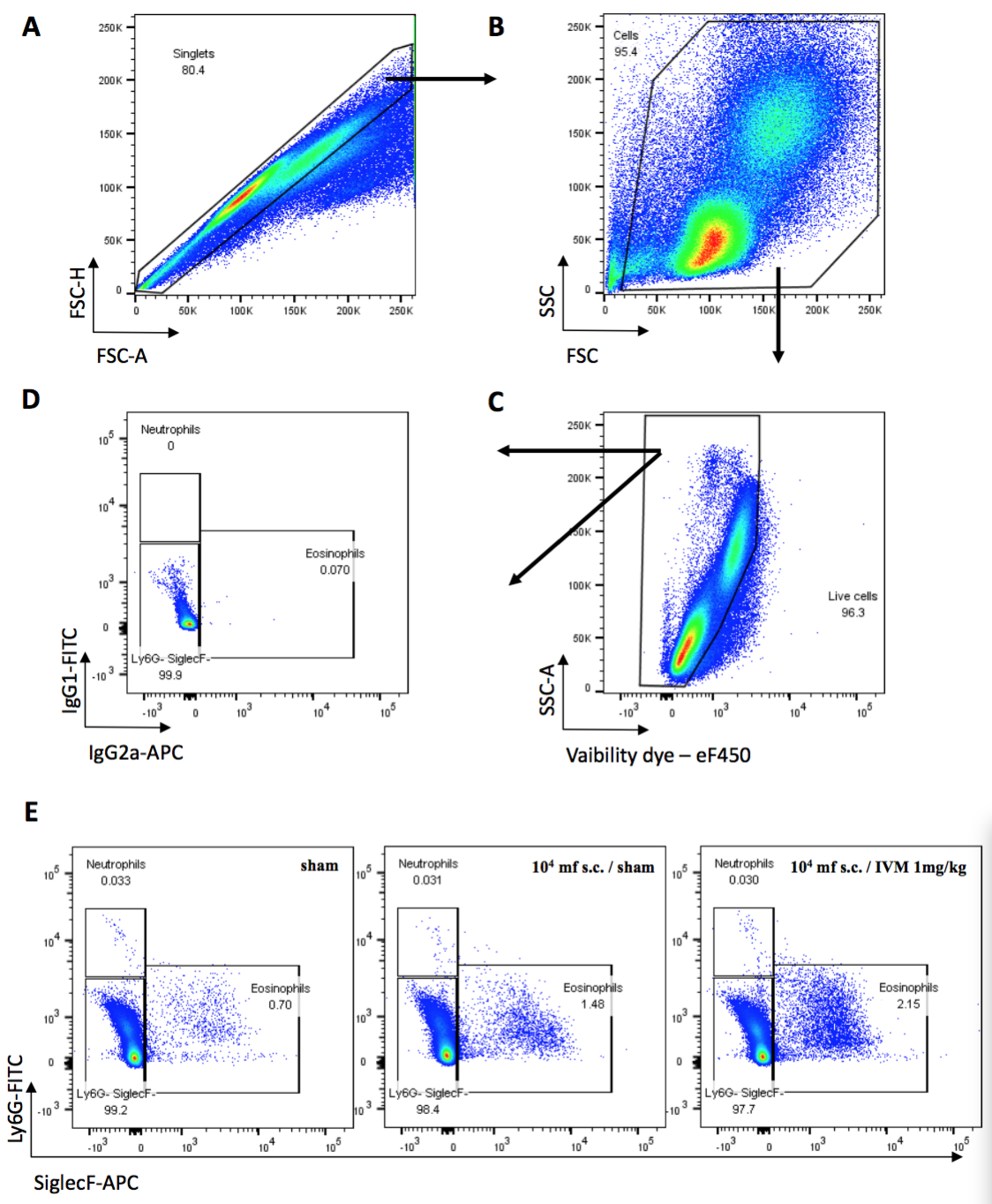


Supplementary Figure 7 : Gating strategy for the immunophenotyping of peritoneal cells. Doublets (A), debris (B) and dead cells (C) were excluded and gating of granulocytes populations were performed using isotype control (D) and specific antibodies (E). Eosinophils were gated as SiglecF^+^Ly6C^-^ and neutrophils as SiglecF^-^Ly6G^+^. (E) representative dot plots for sham, 10^4^ mf s.c. / sham and 10^4^ mf s.c. / IVM 1mg/kg groups, values are reported as % fraction of live cells, n=5-6.

**MZ**

**V**

**RP**

**WP**
