# Supplementary material for: Mouse models of Loa loa
Source: Nat Commun. 2019 Mar 29;10:1429. doi: 10.1038/s41467-019-09442-0 (PMC6441053; doi:10.1038/s41467-019-09442-0)
Supplement: Supplementary file 3 — Reporting Summary [file 41467_2019_9442_MOESM3_ESM.pdf]

nature research

Corresponding author(s): Dr Joseph TURNER

Reporting Summary

Nature Research wishes to improve the reproducibility of the work that we publish. This form provides structure for consistency and transparency in reporting. For further information on Nature Research policies, see Authors & Referees and the Editorial Policy Checklist.

Statistical parameters

When statistical analyses are reported, confirm that the following items are present in the relevant location (e.g. figure legend, table legend, main text, or Methods section).

n/a	Confirmed	
☐	☒	The exact sample size (*n*) for each experimental group/condition, given as a discrete number and unit of measurement
☐	☒	An indication of whether measurements were taken from distinct samples or whether the same sample was measured repeatedly
☐	☒	The statistical test(s) used AND whether they are one- or two-sided<br>*Only common tests should be described solely by name; describe more complex techniques in the Methods section.*
☒	☐	A description of all covariates tested
☐	☒	A description of any assumptions or corrections, such as tests of normality and adjustment for multiple comparisons
☐	☒	A full description of the statistics including central tendency (e.g. means) or other basic estimates (e.g. regression coefficient) AND variation (e.g. standard deviation) or associated estimates of uncertainty (e.g. confidence intervals)
☐	☒	For null hypothesis testing, the test statistic (e.g. *F*, *t*, *r*) with confidence intervals, effect sizes, degrees of freedom and *P* value noted<br>*Give P values as exact values whenever suitable.*
☒	☐	For Bayesian analysis, information on the choice of priors and Markov chain Monte Carlo settings
☐	☒	For hierarchical and complex designs, identification of the appropriate level for tests and full reporting of outcomes
☒	☐	Estimates of effect sizes (e.g. Cohen's *d*, Pearson's *r*), indicating how they were calculated
☐	☒	Clearly defined error bars<br>*State explicitly what error bars represent (e.g. SD, SE, CI)*

Our web collection on statistics for biologists may be useful.

Software and code

Policy information about availability of computer code

Data collection	There were no commercial, open source and custom code used to collect the data in this study. All data was collected from samples and clearly reported in graphs as single data points or central tendancy with variance.
Data analysis	All tests were performed in GraphPad Prism software at a significance level of 5% and significance is indicated P<0.05* P<0.01** P<0.001***. Heatmap analysis was undertaken in Excel using conditional formatting with -10 fold change being color-coded as blue and +10 fold change being reported as red. Data on differential cell counts on either thick smears or immunohistological material were collected in a blinded manner and represent mean counts for 100 leucocytes and 10 quadrants of 120µmx200µm per sample respectively.

For manuscripts utilizing custom algorithms or software that are central to the research but not yet described in published literature, software must be made available to editors/reviewers upon request. We strongly encourage code deposition in a community repository (e.g. GitHub). See the Nature Research guidelines for submitting code & software for further information.

Data

Policy information about availability of data

All manuscripts must include a data availability statement. This statement should provide the following information, where applicable:

- Accession codes, unique identifiers, or web links for publicly available datasets
- A list of figures that have associated raw data
- A description of any restrictions on data availability

All data generated or analysed during this study are included in this published article (and its supplementary information files).

Field-specific reporting

Please select the best fit for your research. If you are not sure, read the appropriate sections before making your selection.

☒ Life sciences ☐ Behavioural & social sciences ☐ Ecological, evolutionary & environmental sciences

For a reference copy of the document with all sections, see nature.com/authors/policies/ReportingSummary-flat.pdf

Life sciences study design

All studies must disclose on these points even when the disclosure is negative.

Sample size	Sample size was based on previous studies of a similar design and double checked with by a power analysis test.
Data exclusions	No data was excluded from the analysis; it indeed helps reflecting natural data heterogeneity.
Replication	Reproducibility of the data was tested by experimental repeats accordingly (as stated in the manuscript) and data were tested for homoscedasticity and distribution before being gathered.
Randomization	For drug treatments, mice were randomly allocated to treatment groups, stratified on time of Loa mf infusion.
Blinding	Investigators involved in parasitological analysis of results were blinded to group allocation during data collection. An Excel spreadsheet has been used to report group allocations and was accessible for investigators who performed mice cull at studies end points. Unblinding of the investigators was done after data analysis.

Reporting for specific materials, systems and methods

Materials & experimental systems

n/a	Involved in the study
☐	☒ Unique biological materials
☐	☒ Antibodies
☒	☐ Eukaryotic cell lines
☒	☐ Palaeontology
☐	☒ Animals and other organisms
☒	☐ Human research participants

Methods

n/a	Involved in the study
☒	☐ ChIP-seq
☐	☒ Flow cytometry
☒	☐ MRI-based neuroimaging

Unique biological materials

Policy information about availability of materials

Obtaining unique materials	The immunodeficient mice used in this study can be purchased from commercial European or Worlwide suppliers. BALB/c, BALB/c RAG2-/-γc-/-, CB.17 SCID, NOD.SCID and NOD.SCIDγc-/- mice of 5-6 weeks of age were purchased from Charles River Europe and BALB/c RAG2-/- mice were kindly provided by Prof Andrew McKenzie (MRC Laboratory of Molecular Biology, Cambridge University, United Kingdom) and by Prof. Dr. Antonius Rolink (Developmental and Molecular Immunology Department of Biomedicine, University of Basel, Switzerland).<br><br>Due to their nature, access to parasites is limiting, with parasite progeny (mf) obtained from splenectomised infected baboons (Papio anubis) that were kept in captivity and infected with the L. loa human strain as previously described. Parasite infective

larvae were derived from wild caught Chrysops silacea via baited traps in a known hyperendemic area. Live or fixed material can be made available upon request

Antibodies

Antibodies used	Our manuscript details the use of an high throuput flow cytometry technique as follow:<br>Plasma cytokines/chemokines levels in mouse plasma were determined using a 32-analyte multiplex cytokine immunoassay based on xMAP technology (MCYTMAG-70K-PX32 kit, Millipore) as per manufacturer's instructions and samples were analyzed on a LX100TM multiplexing instrument (Luminex). Analytes included were: eotaxin, G-CSF, GM-CSF, M-CSF, gamma interferon (IFN-γ), TNF-α, IL-1α, IL-1β, IL-2, IL-3, IL-4, IL-5, IL-6, IL-7, IL-9, IL-10, IL-12 (p40), IL-12 (p70), IL-13, IL-15, IL-17, IP-10, CCL2, CCL3, CCL4, CCL5, CXCL1, CXCL2, CXCL5, CXCL9, leukemia inhibitory factor (LIF), and vascular endothelial growth factor (VEGF).<br><br>In addition, the revised version of the manuscript details an additional FACS analysis performed on samples as follow:<br>Mouse peritoneal cells were collected via a peritoneal cavity wash with 10mL PBS-5% FCS. Cells were subsequently centrifuged (800 rpm, 5min, 4°C) and a Fc blocking step (using α-CD16/32, eBioscience) was performed prior to the application of the following cocktail: viability dye eFluor450 (eBioscience), anti-mouse SiglecF-APC (clone E50-2440, BD Bioscience) and anti-mouse Ly6G-FITC (clone RB6-8C5, eBioscience). All samples were subsequently acquired using a BD LSR II flow cytometer (BD Bioscience) and analysed on FloJo Software. Peritoneal eosinophils were gated on live cells as SiglecFhigh Ly6G- and peritoneal neutrophils on live cells as SiglecF- Ly6Ghigh.
Validation	Validation has been performed following manufacturer's instructions, using standards and quality controls provided in the kit.

Animals and other organisms

Policy information about studies involving animals; ARRIVE guidelines recommended for reporting animal research

Laboratory animals	Species: mouse<br>Sex: male<br>Age: 6-8 weeks at start of the study<br>Strains: BALB/c, BALB/c RAG2-/-, BALB/c RAG2-/-γc-/-, CB.17 SCID, NOD.SCID and NOD.SCIDγc-/-
Wild animals	L. loa microfilariae (mf) were obtained from splenectomised infected baboons (Papio anubis) that were kept in captivity and infected with the L. loa human strain as previously detailed from Wanji et al 2015:<br>"Baboons of both sexes were trapped in different parts of Cameroon according to IPS standard accepted procedures These animals were transported to the animal facilities in the Tropical Medicine Research Station, Kumba, South West Region and quarantined for a period of two months during which they were pre-screened for a panel of natural infections (loiasis, other blood-borne parasites, and intestinal worms). Each animal was observed daily by the veterinary staff to ensure that they were healthy, and any animal found to be ill was immediately given appropriate treatment, both in the quarantine period and during the main study period.<br>The animals were housed individually in large custom built cages that allowed the animals to move about freely and be allowed to display their normal repertoire of locomotor behavior (walking, climbing, running, jumping and swinging) by providing them with vertical climbing surfaces and perches. Horizontal surfaces were also provided to allow them to rest comfortably and perform their social interactions such as sprawling during grooming. The housing facility was well aerated and equipped with a system that provided water ad libitum for each animal. Each baboon's behavior was regularly monitored to identify any indications of poor welfare. Baboons received a diet of food that mimicked their natural diet (leaves, grass, roots, bark, flowers, fruit, lichens, tubers, seeds, mushrooms, corms, and rhizomes). They were also fed a supplement of a nutritionally complete commercial-available diet.<br>The health and well-being of the baboons were regularly assessed during the study by an animal welfare officer who advised on matters such as disease prophylaxis, zoonoses, anesthe- sia, and methods of humane euthanasia and provision of health certificates. All measures were taken to minimize suffering during capture, captivity and experimentation. Health screening of workers in contact with the baboons was performed regularly to prevent animal losses from diseases transmitted from humans to baboons as well as zoonotic transmission of disease from baboons to workers."<br><br>Ethical and administrative clearances for the use of baboons in this study were obtained from the Ministry of Scientific Research and Innovation of Cameroon (Research permit #028/MINRESI/B00/C00/C10/C12) and the Animal Care Committee at REFOTDE. Procedures adhered to the NIH Guide for the Care and Use of Laboratory Animals.<br><br>The study also involved wild Chrysops silacea (a fly, vector of the parasite) being captured via baited traps in a known hyperendemic area
Field-collected samples	Flies were dissected to allow release of any L. loa L3 infective stages. Infective doses of 100 to 200 L3 per 200µL medium (DMEM + 10% FCS) were loaded in 25G 1mL syringes and subcutaneously injected into mice.

Flow Cytometry

Plots

Confirm that:

☒ The axis labels state the marker and fluorochrome used (e.g. CD4-FITC).

☒ The axis scales are clearly visible. Include numbers along axes only for bottom left plot of group (a 'group' is an analysis of identical markers).

☒ All plots are contour plots with outliers or pseudocolor plots.

☒ A numerical value for number of cells or percentage (with statistics) is provided.

Methodology

Sample preparation	Luminex: Mice sera were collected at readout and 50µL neat serum per mouse were used.<br>FACS: Mouse peritoneal cells were collected via a peritoneal cavity wash with 10mL PBS-5% FCS. Cells were subsequently centrifuged (800 rpm, 5min, 4°C) and a Fc blocking step (using α-CD16/32, eBioscience) was performed prior to the application of the antibodies cocktail.
Instrument	Luminex: Samples were analyzed on a LX100TM multiplexing instrument.<br>FACS: All samples were subsequently acquired using a BD LSR II flow cytometer (BD Bioscience).
Software	Luminex: xPONENT 3.1 software was used for the data collection and Excel was subsequently used for an heatmap analysis using conditional formatting with -10 fold change being color-coded as blue and +10 fold change being reported as red.<br>FACS: All samples were analysed on FloJo Software. Peritoneal eosinophils were gated on live cells as SiglecFhigh Ly6G- and peritoneal neutrophils on live cells as SiglecF- Ly6Ghigh.
Cell population abundance	Luminex: Sufficient data was collected for analysis and this sufficiency was ensured by Luminex run parameters with either 60 seconds acquisition per sample or 50 events per bead (according to manufacturer's instructions).<br>FACS: Whole samples were acquired to allow the maximum number of events to be recorded per targeted cell population.
Gating strategy	Luminex: There was no gating strategy used as this high throughput assay directly returns analyte concentration in each sample. Acquisition parameters were displayed and investigators ensured that sufficient information was gathered for each sample.<br>FACS: gating strategy is detailed in Supp Figure 6 and is based on exclusion of doublets and dead cells.

☒ Tick this box to confirm that a figure exemplifying the gating strategy is provided in the Supplementary Information.
