# Supplementary material for: Mouse models of Loa loa
Source: Nat Commun. 2019 Mar 29;10:1429. doi: 10.1038/s41467-019-09442-0 (PMC6441053; doi:10.1038/s41467-019-09442-0)
Supplement: Supplementary file 4 — Description of Additional Supplementary Files [file 41467_2019_9442_MOESM4_ESM.pdf]

Description of Additional Supplementary Files

File Name: Supplementary Movie 1
Description: Loa males and females in ex vivo cultures derived from a single NOD.SCIDγc -/- mouse, 5 months post-infection

File Name: Supplementary Movie 2
Description: Ruptured female Loa worm and liberated uterine-released contents, including motile mf, in ex vivo culture derived from a NOD.SCIDγc -/- mouse, 5 months post-infection

File Name: Supplementary Movie 3
Description: Migrating Loa adult worm under the skin of a BALB/c RAG2-/- mouse seven days post-implantation

File Name: Supplementary Movie 4
Description: Motile Loa adult worm within subcutaneous tissue at necropsy, one month post-implantation of a BALB/c RAG2-/- mouse
